# Knowledge, attitudes, and practices on infection prevention and control among healthcare workers in Rohingya refugee camp

**DOI:** 10.1016/j.ijregi.2025.100750

**Published:** 2025-09-07

**Authors:** Md. Atiar Rahman Mondol, Md. Monir Hossain Shimul, Sikder Masud Raihan, Saimum Arafat Pantho, Salamat Khandker

**Affiliations:** 1 Department of Public Health, Daffodil International University, Dhaka, Bangladesh; 2 Islami Bank Central Hospital, Dhaka, Bangladesh; 3 Department of Anesthesiology, Labaid Specialized Hospital, Dhaka, Bangladesh

**Keywords:** Infection prevention and control (IPC), Knowledge, attitude, practice (KAP), Healthcare workers, Refugee camp, FDMN, Cross-sectional study

## Abstract

**Objectives:**

The forcibly displaced Myanmar nationals (FDMN) camp in Cox’s Bazar, Bangladesh, is highly vulnerable to infectious disease outbreaks due to overcrowding and limited healthcare infrastructure. This study aimed to assess the knowledge, attitudes, and practices (KAP) related to infection prevention and control (IPC) among healthcare workers (HCWs) in the FDMN camp to identify gaps and inform targeted interventions.

**Methods:**

A cross-sectional study was conducted between September 2023 and July 2024 among 401 HCWs employed in Primary Health Care Centers, Health Posts, Community Clinics, and Upazila Health Complexes within the FDMN camp. Respondents were randomly selected from the healthcare workforce list of the local civil surgeon’s office. Data were collected using a semi-structured, pretested questionnaire. The questionnaire covered sociodemographics and a KAP survey was developed based on the World Health Organization IPC protocol. Descriptive statistics, chi-square tests, and association analyses were performed using SPSS version 22, with significance set at *P* <0.05.

**Results:**

Among 401 healthcare workers, 60.6% were female and 39.4% were male. Overall, 71.8% had good IPC knowledge, 64.4% showed positive attitudes, and 85.5% demonstrated good practices. Knowledge was significantly associated with age, experience, job station, and training (all *P* <0.01), while attitudes were influenced by experience and training (*P* <0.01). Practice was significantly associated with sex, experience, job station, and training (all *P* <0.01).

**Conclusions:**

Healthcare workers in the FDMN camp showed good IPC knowledge and practices, though attitudes varied. Training significantly improved all KAP domains. Strengthening NGO-led training, supervision at PHCCs, and routine KAP monitoring is vital for sustainable infection control in refugee settings.

## Highlights

• 71.8% of healthcare workers showed good infection prevention and control (IPC) knowledge in a high-risk refugee setting.
• IPC training significantly improved knowledge, attitude, and practice (*P* <0.01).
• Practice quality was influenced by sex, experience, job station, and training (*P* <0.01).
• Workers with less than 2 years of experience had poorer IPC attitudes and practices.
• Strong evidence supports the need for regular IPC training in humanitarian contexts.

## Introduction

Infection prevention and control (IPC) has been increasingly acknowledged in the wake of global health emergencies. Outbreaks of Ebola, SARS, and the COVID-19 pandemic exposed systemic vulnerabilities in IPC measures, even in well-resourced health systems [1]. At its core, IPC is a key strategy to curb the transmission of healthcare-associated infections (HAIs) and safeguard clinical outcomes [2]. The World Health Organization (WHO) estimates that in low- and middle-income countries (LMICs), the burden of HAIs is up to 20 times higher than in high-income countries, posing a considerable threat to both health system performance and patient safety [3,4].

Overcrowding, inadequate sanitation, limited access to clean water, and insufficient supplies of personal protective equipment and medical resources significantly compromise IPC compliance and effectiveness [5,6]. The forcibly displaced Myanmar nationals (FDMN) residing in the refugee camps of Cox’s Bazar, Bangladesh, represent one of the world’s largest stateless populations. These camps are characterized by high population density, poor infrastructure, and constrained healthcare systems, all of which create ideal conditions for the rapid spread of infectious diseases [7,8]. This creates a heightened risk of communicable disease outbreaks, necessitating stringent IPC measures.

Healthcare workers’ knowledge, attitudes, and practices (KAP) regarding IPC are often influenced by limited training, lack of resources, and overwhelming workloads [[9], [10], [11], [12]]. Studies from LMICs show that knowledge levels in Ethiopia and Ghana ranged between 57% and 60%, respectively, while in Saudi Arabia, they were as high as 80% [[9], [10], [11]]. These discrepancies underscore the urgent need for context-specific assessments to better understand and address local IPC challenges. In the FDMN refugee camp, there remains a dearth of focused research evaluating the KAP of healthcare professionals. Studies in Bangladesh have heightened challenges in IPC during COVID-19 [12], while Rohingya camp assessments revealed poor water, sanitation, and hygiene (WASH) conditions and infection risks [13,14]. These findings underscore the urgency of assessing HCWs’ IPC in the FDMN context.

Bangladesh faces enormous strain on its healthcare infrastructure, particularly in resource-limited settings like refugee camps. The scarcity of trained personnel, coupled with limited monitoring and evaluation of IPC adherence, aggravates the potential for HAIs to proliferate unchecked [12,15]. Moreover, linguistic barriers, cultural diversity, and the transient nature of both healthcare providers and refugee populations further hinder the delivery of standardized infection control training and services [8,16].

This study seeks to assess the level of KAP regarding IPC among healthcare workers serving in the FDMN refugee camps. By identifying key gaps and strengths, the study aims to design targeted interventions and training modules that will enhance IPC capacity in these vulnerable settings. Specifically, this study aimed to (i) assess HCWs’ IPC knowledge, attitudes, and practices in the FDMN camp and (ii) identify factors associated with KAP outcomes, including training, job placement, and experience.

This research contributes to a growing body of evidence needed to reinforce IPC in refugee settings and offers actionable insights for health policymakers, humanitarian organizations, and frontline workers to protect both providers and patients in crisis-prone environments.

## Methodology

### Study design

This study employed a descriptive cross-sectional survey design to assess the KAP related to IPC among HCWs operating in the FDMN refugee camp in Cox’s Bazar, Bangladesh. The study spanned from September 2023 to July 2024.

### Study area

The study was carried out across Primary Health Care Centers (PHCC), Health Posts, Community Clinics (CC), and Upazila Health Complexes (UHC) within the FDMN refugee camps. A PHCC refers to a relatively larger healthcare facility operating in the camp environment, staffed by medical doctors, nurses, midwives, and laboratory personnel, that offers outpatient services, maternal and child healthcare, and limited emergency management. A health post represents a smaller unit of healthcare delivery, generally managed by nurses or medical assistants, and is responsible for providing basic curative services, health education, and referrals. A CC is a grassroots-level healthcare facility established by the Government of Bangladesh to serve rural populations, including those living in refugee settlements. Each clinic caters to approximately 6000 people and is typically operated by a Community Healthcare Provider (CHCP), delivering services such as immunizations, antenatal care, and treatment for common illnesses. A UHC serves as the main sub-district-level hospital that offers a wide range of outpatient and inpatient services, including diagnostics, minor surgery, and emergency care. It is usually equipped with a multidisciplinary team of health professionals and administrative support staff.

### Study population

The target population comprised healthcare workers involved in direct patient care or facility-level infection control operations. These included medical doctors, nurses, midwives, medical technologists, medical assistants, community health workers (CHWs), community health volunteers (CHVs), and other clinical support staff.

### Sample size and sampling technique

The minimum required sample size was calculated using the formula n = Z^2^pq/d^2^, assuming a 95% confidence level (z = 1.96), a proportion (p) of 0.5 (50%), and a margin of error (d) of 5%. This resulted in a sample size of 384, which was increased to 401 to account for non-response and enhance representativeness. We collected a list of health sector workers from the office of the civil surgeon. From this list, we randomly selected 32 medical doctors, 68 nurses, 73 midwives, 32 medical assistants, 80 CHWs & volunteers, and 116 clinical support staff (facility in-charges, technologists, administrative staff, vaccinators, ambulance drivers, and cleaners).

### Inclusion and exclusion criteria

HCWs currently employed in the FDMN camp and directly involved in patient care or infection control activities were eligible to participate. Exclusion criteria included administrative staff not engaged in clinical services, temporary HCWs without regular IPC exposure, and any HCW not working within the camp at the time of data collection.

### Data collection tools

Data were collected using a pretested questionnaire designed to assess IPC-related KAP that was developed based on the WHO IPC protocol and validated literature [1,2]. The instrument included both closed- and open-ended questions. The questionnaire included sections on sociodemographics, knowledge indicators, attitudinal statements, and reported practices. To categorize the levels of KAP, Bloom’s cut-off point was used as the judgment criterion. For each domain, responses were scored and converted to percentage values. Participants who scored 80% or higher were considered to have ‘good’ knowledge, attitude, or practice; those scoring between 60% and 79% were categorized as ‘moderate’; and those scoring below 60% were classified as having ‘poor’ levels (Bloom, 1956; adapted for KAP surveys [9]). Knowledge scores were derived from correct responses to factual IPC questions. Attitudes were assessed using a Likert scale, while practice was evaluated through self-reported frequency of IPC-related behaviors.

### Study variables

The dependent variables in the study were the levels of KAP regarding IPC among HCWs. Independent variables included age, gender, job designation, length of professional experience, job station, type of organization, and prior IPC training.

### Data collection and quality control

Data were collected by trained field researchers through face-to-face interviews under the close supervision of the principal investigator. The questionnaire was pretested among healthcare workers in similar healthcare settings outside the study area. Double data entry and random quality checks were performed during data input into SPSS version 22 to minimize errors and ensure consistency. Cronbach’s alpha was calculated at 0.81 for knowledge, 0.79 for attitudes, and 0.83 for practices, indicating acceptable internal consistency.

### Data analysis

Descriptive statistics were computed to summarize demographic characteristics and KAP levels. Inferential statistics, including chi-square tests, were used to examine associations between KAP outcomes and sociodemographic factors. A significance level of *P* <0.05 and 95% confidence intervals (CIs) were applied to all statistical tests.

### Ethical clearance

This study received ethical approval from the Research Ethics Committee (REC) of the Faculty of Health and Life Sciences, Daffodil International University (Approval No. FHLSREC/DIU/2023/SMIG-122). All study procedures adhered to the principles of the Declaration of Helsinki.

### Human ethics and consent to participate

Participation in this study was voluntary. Informed written consent was obtained from all participants after the purpose, procedures, potential risks, and benefits of the study were explained. Participants were assured of confidentiality, and no personal identifiers were used in data analysis or reporting. Respondents were also informed of their right to withdraw at any point without consequence.

## Results

Table 1 shows that the healthcare workers of the FDMN refugee camp is largely composed of younger individuals, with 97.3% aged between 20 and 40 years (mean age: 28.3 ± 4.7 years), and includes a higher proportion of females (60.6%) than males (39.4%). Most respondents are based at PHCCs (59.4%) and health posts (35.2%), while fewer are located in CCs (4.2%) and UHCs (1.2%).

**Table 1:** Sociodemographic and professional characteristics of healthcare workers in the forcibly displaced Myanmar nationals Refugee Camp (n = 401).

Variable	Category	Frequency (n)	Percentage (%)
**Age**	20-40	390	97.3
	41-60	11	2.7
	*Mean ± SD*	*28.3 ± 4.7*	
**Sex**	Male	158	39.4
	Female	243	60.6
**Job station**	Primary health care center	238	59.4
	Health post	141	35.2
	Community clinic	17	4.2
	Upazila health complex	5	1.2
**Current designation**	Nurse	68	17.0
	Midwife	73	18.0
	Community health worker/Community health volunteer	80	20.0
	Doctor	32	8.0
	Medical assistant	32	8.0
	Facilities in-charge, medical technologists, administrative staff, vaccinator, ambulance driver, cleaner.	116	29.0
**Professional experience**	<2 years	277	69.1
	3-5 years	111	27.7
	>5 years	13	3.2
**Received IPC training**	Yes	278	69.3
	No	123	30.7
**Idea about IPC**	Yes	363	90.5
	No	38	9.5

IPC, infection prevention and control.

Regarding professional roles, CHWs/CHVs form the single largest category (20%), closely followed by midwives (18.0%) and nurses (17%). Other roles, including administrative and support staff, represent 29% of the respondents, while doctors and medical assistants each make up 8%. In terms of experience, a substantial majority (69.1%) of the participants have less than 2 years of service, with 27.7% having 3-5 years, and only 3.2% reporting over 5 years of professional experience.

Encouragingly, 69.3% of healthcare workers reported having received formal training in IPC, and an even greater proportion (90.5%) expressed awareness of IPC practices.

Table 2 highlights that a significant majority of healthcare workers in the FDMN refugee camp demonstrated good knowledge (71.8%), while 15.5% had poor knowledge regarding IPC. Attitudinally, 64.4% exhibited a good attitude toward IPC, although 7.2% still held poor attitudes, suggesting a need for targeted behavioral interventions. Most notably, a statistically significant proportion (85.5%) reported good IPC practices (*P* <*0.05*), reinforcing the positive correlation between knowledge, attitude, and adherence to best practices.

**Table 2:** Distribution of knowledge, attitude, and practice levels among respondents (n = 401).

Level	Knowledge (%)	Attitude (%)	Practice (%)
**Good**	288 (71.8)	259 (64.6)	343 (85.5)
**Moderate**	51 (12.7	114 (28.4)	48 (12.0)
**Poor**	62 (15.5)	28 (7.0)	10 (2.5)

Table 3 demonstrates that a statistically significant association was observed between knowledge level and age (χ² = 9.76, *P* <0.01), where younger workers aged 20–40 showed greater knowledge levels. Years of experience also had a strong influence on knowledge, with those having less than 2 years showing lower knowledge compared to more experienced groups (χ² = 30.33, *P* <0.01). Similarly, job station played a role in knowledge distribution, with workers from PHCC exhibiting lower knowledge scores compared to those from health posts or other settings (χ² = 46.55, *P* <0.01). The most striking association was found with IPC training, where trained participants overwhelmingly had good knowledge (67.3%) compared to their untrained counterparts, where poor knowledge predominated (15.2%) (χ² = 308.7, *P* <0.01). However, no statistically significant difference was noted by sex (*P* = 0.17), indicating that knowledge levels were fairly distributed between male and female respondents.

**Table 3:** Association between sociodemographic factors and level of knowledge on infection prevention and control among the respondents (n = 401).

Variable		Level of knowledge			χ² value	*P*-value
		Good n (%)	Moderate n (%)	Poor n (%)		
**Sex**	Male	110 (27.4)	26 (6.5)	22 (5.5)	3.43	0.17
	Female	178 (44.4)	25 (6.2)	40 (10.0)		
**Age**	20-40	282 (70.3)	46 (11.5)	62 (15.5)	9.76	<0.01
	41-60	6 (1.5)	5 (1.2)	0 (0.0)		
**Experience**	<2 years	177 (44.1)	42 (10.5)	58 (14.5)	30.33	<0.01
	3-5 years	98 (24.4)	9 (2.2)	4 (1.0)		
	>5 years	13 (3.2)	0 (0.0)	0 (0.0)		
**Job station**	Primary health care center	146 (36.4)	35 (8.7)	57 (14.2)	46.55	<0.01
	Health post	122 (30.4)	15 (3.7)	4 (1.0)		
	Community clinic	15 (3.7)	1 (0.2)	1 (0.2)		
	Upazila health complex	5 (1.2)	0 (0.0)	0 (0.0)		
**Received training**	Yes	270 (67.3)	7 (1.7)	1 (0.2)	308.7	<0.01
	No	18 (4.5)	44 (11.0)	61 (15.2)		

Table 4 illustrates that the attitude level was not significantly associated with sex (χ² = 2.47, *P* = 0.29) or age group (χ² = 4.42, *P* = 0.11), or job station (*P* = 0.22). However, experience and training received on IPC were statistically significant predictors of attitude. Healthcare workers with less than 2 years of experience were more likely to have moderate or poor attitudes compared to those with longer experience (χ² = 33.84, *P* < 0.01). Moreover, training had a highly significant influence on attitude—58.1% of trained participants had a good attitude versus only 6.5% among those untrained, while the proportion of poor attitudes was much higher among untrained individuals (6.0% vs 1.0%) (χ² = 153.67, *P* <0.01).

**Table 4:** Association between sociodemographic variables and attitude toward infection prevention and control among the respondents (n = 401).

Variable		Level of attitude			χ² value	*P*-value
		Good n (%)	Moderate n (%)	Poor n (%)		
**Sex**	Male	108 (26.9)	38 (9.5)	12 (3.0)	2.47	0.29
	Female	151 (37.7)	76 (19.0)	16 (4.0)		
**Age**	20-40	254 (63.3)	108 (26.9)	28 (7.0)	4.42	0.11
	41-60	5 (1.2)	6 (1.5)	0 (0.0)		
**Experience**	<2 years	157 (39.7)	94 (23.4)	26 (6.5)	33.84	<0.01
	3-5 years	89 (22.2)	20 (5.0)	2 (0.5)		
	>5 years	13 (3.2)	0 (0.0)	0 (0.0)		
**Job station**	Primary health care center	147 (36.7)	76 (19.0)	15 (3.7)	8.15	0.22
	Health post	95 (23.7)	35 (8.7)	11 (2.7)		
	Community clinic	12 (3.0)	3 (0.7)	2 (0.5)		
	Upazila health complex	5 (1.2)	0 (0.0)	0 (0.0)		
**Received training**	Yes	233 (58.1)	41 (10.2)	4 (1.0)	153.67	<0.01
	No	26 (6.5)	73 (18.2)	24 (6.0)		

Table 5 presents a statistically significant association with sex (χ² = 11.68, *P* <0.01), where female participants exhibited a higher proportion of good, moderate, and poor practices compared to males. Professional experience also significantly influenced practice levels (χ² = 28.25, *P* <0.01); those with less than 2 years of experience showed more moderate or poor practices than those with more experience.

**Table 5:** Association between sociodemographic variables and infection prevention and control practice among the respondents (n = 401).

Variable		Level of practice			χ² value	*P*-value
		Good n (%)	Moderate n (%)	Poor n (%)		
**Sex**	Male	146 (36.4)	11 (2.7)	1 (0.2)	**11.68**	**<0.01**
	Female	197 (49.1)	37 (9.2)	9 (2.2)		
**Age**	20-40	333 (83.0)	48 (12.0)	9 (2.2)	3.89	0.14
	41-60	10 (2.5)	0 (0.0)	1 (0.2)		
**Experience**	<2 years	222 (55.4)	46 (11.5)	9 (2.2)	**28.25**	**<0.01**
	3-5 years	108 (26.9)	2 (0.5)	1 (0.2)		
	>5 years	13 (3.2)	0 (0.0)	0 (0.0)		
**Job station**	Primary health care center	184 (45.9)	45 (11.2)	9 (2.2)	**40.77**	**<0.01**
	Health post	138 (34.4)	2 (0.5)	1 (0.2)		
	Community clinic	16 (4.0)	1 (0.2)	0 (0.0)		
	Upazila health complex	5 (1.2)	0 (0.0)	0 (0.0)		
**Received training**	Yes	275 (68.6)	3 (0.7)	0 (0.0)	**130.35**	**<0.01**
	No	68 (17.0)	45 (11.2)	10 (2.5)		

Additionally, job station had a meaningful impact (χ² = 40.77, *P* <0.01), with workers stationed at PHCCs demonstrating more moderate and poor practice levels compared to those at health posts or CCs. The most significant finding was related to IPC training, where none of the trained participants exhibited poor practice, while 2.5% of untrained participants did (χ² = 130.35, *P* <0.01). On the other hand, age was not significantly associated with practice levels (*P* = 0.14), indicating consistent IPC practice across age groups.

## Discussion

The current study examined the KAP concerning IPC among healthcare workers in the FDMN refugee camp in Cox’s Bazar, Bangladesh. This population represents a particularly vulnerable group given the complex healthcare challenges associated with forced displacement, overcrowded living conditions, limited infrastructure, and elevated risk of communicable disease outbreaks. The findings of this study align with existing global concerns regarding IPC in humanitarian settings and shed light on both the progress and the gaps in the refugee healthcare context.

A key strength observed was the relatively high level of IPC knowledge and practice among participants, particularly among younger healthcare workers and those who received training. This aligns with WHO guidelines that emphasize the necessity of structured IPC programs and routine training to prevent HAIs in both acute care and emergency settings [1,4]. The elevated awareness and adherence to IPC protocols among trained personnel in this study reflect the impact of capacity-building efforts and underscore the importance of sustaining these initiatives, especially in high-risk, resource-constrained environments [17].

Comparable trends have been observed in similar global contexts. In Ethiopia and Ghana, for instance, studies have demonstrated a positive correlation between IPC knowledge and professional training, although the baseline knowledge levels were comparatively lower than those found in this study [5,6]. Similarly, healthcare workers in Jordanian refugee camps also displayed substantial IPC knowledge, particularly when training had been consistently provided [18]. However, nearly one-third of participants still demonstrated poor or moderate knowledge, highlighting persistent gaps. These parallels confirm that structured training, regardless of context, remains an essential determinant in improving IPC compliance among frontline workers.

The findings also demonstrate that professional experience and workplace designation significantly influence both attitude and practice. PHCC staff showed weaker knowledge scores compared to those in other facilities, yet their reported IPC practices were stronger. This apparent discrepancy may be explained by organizational enforcement, better supply availability, or closer supervisory oversight at PHCCs. These contextual factors likely contributed to more consistent practice despite lower knowledge levels. This observation resonates with research conducted in Uganda and Ethiopia, where workers in better-resourced units showed greater adherence to IPC guidelines than those in peripheral or under-resourced facilities [19,20].

Attitudinal trends among healthcare workers in the FDMN refugee camp, while moderately positive overall, indicate room for improvement. Although a substantial proportion expressed good attitudes toward IPC, a notable fraction held moderate or indifferent views. Previous studies confirm that occupational stress and cultural context affect IPC attitudes, with evidence from Nigeria and Kenya showing more polarized attitudes by gender and age [21,22]. However, comparable findings have also been reported in Rohingya camps in Cox’s Bazar [8] and in Syrian and Lebanese refugee camps [13,23], highlighting that refugee-specific conditions further shape IPC-related beliefs. In contrast, our study found no significant associations with sex or age, suggesting that contextual factors such as training and experience may override demographic determinants in shaping attitudes.

Globally, IPC implementation in refugee settings remains a significant challenge. The FDMN camp is no exception. Issues such as overcrowding, insufficient sanitation facilities, and cultural and linguistic barriers hinder consistent IPC practices [2,8]. These systemic challenges are echoed in reports from other refugee crises, such as in South Sudan and Lebanon, where, despite knowledge and willingness, infrastructural limitations act as a barrier to effective practice [23,24]. The WHO has long emphasized that without an enabling environment, even the best-trained workforce may fall short in implementing IPC protocols effectively [1,25].

This study directly addresses its stated objectives by exploring the sociodemographic profile of healthcare workers, evaluating the extent of their IPC knowledge, measuring their attitudes and behaviors, and identifying the associations between training, age, experience, and job station. The hypothesis that training positively influences KAP outcomes is strongly supported by the data. The significant differences between trained and untrained workers affirm the hypothesis and highlight the critical need for regular and standardized IPC training programs. These results also reinforce findings from studies conducted in diverse low-resource settings, including Bangladesh and conflict-affected regions of the Middle East [12,26].

Despite the promising outcomes, the cross-sectional nature of the study restricts causal inferences; thus, while associations are clear, temporal relationships cannot be confirmed. The reliance on self-reported data may introduce social desirability or recall bias, which may lead participants to overstate their adherence to IPC protocols. Additionally, this study focuses solely on one refugee camp, limiting the generalizability of findings to other settings or countries with different health infrastructure or cultural contexts. Moreover, the specific content and delivery methods of IPC training were not assessed, which limits understanding of which aspects of training are most effective.

Future research should consider longitudinal designs to assess how IPC knowledge and practices evolve over time with ongoing training or infrastructural improvements. Further investigation is also needed into the psychosocial and environmental factors that influence attitudes, particularly in high-stress humanitarian settings. Multi-site studies comparing IPC practices across refugee camps could provide deeper insights into best practices and help establish global benchmarks for humanitarian IPC preparedness.

In light of the findings, we recommend expanding and institutionalizing IPC training for all healthcare workers in refugee settings, with particular emphasis on newly recruited staff and those in under-resourced facilities. Policies should mandate periodic refresher training, while monitoring systems should be implemented to ensure adherence to IPC protocols across all service points. These measures will contribute to a safer healthcare environment for both workers and patients and reduce the risk of preventable infections in displaced populations.

## Conclusion

This study comprehensively assessed the KAP of healthcare workers regarding IPC in the FDMN refugee camp. The findings confirmed the study’s hypothesis that structured training significantly enhances IPC-related KAP outcomes. Younger and less experienced workers, particularly those stationed at PHCCs and who had received training, demonstrated higher compliance and better understanding of IPC protocols. Despite commendable performance in practice and knowledge, attitudinal gaps remain, highlighting the need for culturally tailored interventions. To mitigate infection risks in refugee healthcare settings, consistent training, infrastructural support, and monitoring should be prioritized.

## Declaration of competing interest

The authors have no competing interests to declare.
